# The multifaceted roles of lysosomes in cancer

**DOI:** 10.1002/1878-0261.70349

**Published:** 2026-11-04

**Authors:** Jia Li, Kevin M. Ryan

**Affiliations:** ^1^ Cancer Research UK Scotland Institute Glasgow UK; ^2^ School of Cancer Sciences University of Glasgow UK

**Keywords:** cancer, lysosome, membrane contact sites, metabolism, nutrient sensing, tumour immunity

## Abstract

Cancer cells proliferate rapidly and require sustained supplies of nutrients and biosynthetic precursors. Lysosomes are more than terminal degradation sites: they recycle amino acids, sugars, lipids, nucleosides and ions, while also coordinating nutrient sensing, organelle communication and immune regulation. Consequently, lysosomal capacity can become selectively advantageous in tumours by linking nutrient salvage with growth control, organelle communication and inflammatory signalling. Enhanced lysosomal function can support tumour growth, invasion and therapy resistance. Here, we review four cancer‐relevant dimensions of lysosomal capacity: catabolism and transport; mTORC1‐ and AMPK‐linked nutrient sensing; membrane contact sites with the ER, mitochondria, and Golgi; and immune regulation spanning innate and adaptive responses and inflammatory control. These functions are context dependent: lysosomal quality control can restrain tumour initiation, whereas established cancers often exploit increased lysosomal capacity to support proliferation, invasion, metastasis and therapy resistance. Finally, we discuss lysosome‐state biomarkers and lysosome‐targeted strategies for patient stratification and rational combination therapy.

## Graphical abstract

Lysosomes support cancer cell adaptation by integrating macromolecule degradation and nutrient recycling with nutrient sensing, inter‐organelle communication and immune regulation. These interconnected functions can promote tumour growth, stress tolerance and therapy resistance, while also creating context‐dependent vulnerabilities that may be exploited for biomarker development and targeted combination therapies.

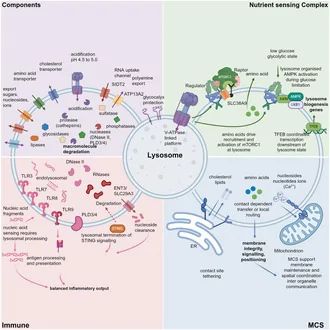

**Abbreviations**
AMPK: AMP‐activated protein kinase
APP: amyloid precursor protein
ATRAID: all‐trans retinoic acid‐induced differentiation factor
BMP: bis(monoacylglycero)phosphate (lysobisphosphatidic acid)
cGAS: cyclic GMP‐AMP synthase
CLN3: ceroid lipofuscinosis neuronal protein 3
CLN5: ceroid lipofuscinosis neuronal protein 5
DNase II: deoxyribonuclease II
DRAM1: DNA damage‐regulated autophagy modulator 1
dsRNA: double‐stranded RNA
ENT3: equilibrative nucleoside transporter 3 (SLC29A3)
ER: endoplasmic reticulum
ESCRT: endosomal sorting complexes required for transport
FLCN: folliculin
GATOR1: GAP activity towards Rags complex 1
GLMP: glycosylated lysosomal membrane protein
GLUT8: glucose transporter 8 (SLC2A8)
GPD: glycerophosphodiester
IL‐1β: interleukin 1 beta
KICSTOR: KPTN‐ITFG2‐C12orf66‐SZT2 complex
LAMP: lysosome‐associated membrane protein
LKB1: liver kinase B1
LSD: lysosomal storage disorder
MCS: membrane contact site
MFSD1: major facilitator superfamily domain‐containing 1
MHC: major histocompatibility complex
mtDNA: mitochondrial DNA
mTORC1: mechanistic target of rapamycin complex 1
mtRNA: mitochondrial RNA
NKG7: natural killer cell granule protein 7
NLRP3: NLR family pyrin domain‐containing 3
NPC1: Niemann–Pick C1 protein
NPC2: Niemann–Pick C2 protein
ORP1L: oxysterol‐binding protein‐related protein 1‐like
ORP5: oxysterol‐binding protein‐related protein 5
PAC: proton‐activated chloride channel
PHT1: peptide histidine transporter 1 (SLC15A4)
PHT2: peptide histidine transporter 2 (SLC15A3)
PI (3,5) P2: phosphatidylinositol 3,5‐bisphosphate
PI (4,5) P2: phosphatidylinositol 4,5‐bisphosphate
PLA2G15: phospholipase A2 group XV
PLBD1: phospholipase B domain‐containing 1
PLD3: phospholipase D3
PLD4: phospholipase D4
PROTAC: proteolysis‐targeting chimaera
Rag: Rag GTPase
Ragulator: Ragulator complex
Rheb: Ras homologue enriched in brain
RNase: ribonuclease
SAMTOR: S‐adenosylmethionine sensor upstream of mTORC1
SIDT2: SID1 transmembrane family member 2
SLC: solute carrier
SLC37A3: solute carrier family 37 member 3
SLC38A9: solute carrier family 38 member 9
SLC7A11: solute carrier family 7 member 11
SNARE: soluble NSF attachment protein receptor
SPNS1: spinster homologue 1
STIM1: stromal interaction molecule 1
STING: stimulator of interferon genes
SYT7: synaptotagmin 7
TFE3: transcription factor E3
TFEB: transcription factor EB
TLR: Toll‐like receptor
TMEM175: transmembrane protein 175
tRNA: transfer RNA
TRPML1: transient receptor potential mucolipin 1
UNC93B: UNC93B1 protein
VAP‐A/B: VAMP‐associated protein A and B
v‐ATPase: vacuolar‐type H+ ATPase
VDAC1: voltage‐dependent anion channel 1

## 1 Introduction

Cancer cells develop within environments that impose substantial and changing demands on cellular homeostasis. Rapid oncogenic growth increases the requirement for nutrients, energy and biosynthetic precursors. At the same time, cancer cells need to adapt to immune pressure, remodel the extracellular matrix during invasion, survive detachment and metastatic dissemination, and tolerate damage caused by chemotherapy, radiotherapy and targeted treatments. These conditions create strong selective pressure for pathways that recycle cellular material, acquire extracellular nutrients, maintain organelle integrity and rapidly adjust growth and stress responses. Lysosomes are particularly well placed to support these adaptations and have therefore emerged as important regulators of tumour cell fitness and tumour–immune interactions.

Lysosomes are membrane‐bound acidic organelles that function as major degradative and recycling compartments of the cell. Their lumen is maintained at approximately pH 4.5–5.0 by the vacuolar‐type H^+^‐ATPase (v‐ATPase), providing the acidic environment required for hydrolases that degrade proteins, lipids, glycans, and nucleic acids. The limiting lysosomal membrane is protected by highly glycosylated lysosome‐associated membrane proteins (LAMPs) and contains transporters and ion channels that export degradation products and maintain ionic homeostasis.

Cargo reaches lysosomes through several interconnected trafficking pathways. During macroautophagy, portions of the cytoplasm, including proteins and organelles, are enclosed within double‐membrane autophagosomes that subsequently fuse with lysosomes, allowing their contents to be degraded and recycled. Selective forms of autophagy use dedicated recognition mechanisms to target particular cargo, such as damaged mitochondria or protein aggregates, for lysosomal degradation. Endocytosis and receptor trafficking deliver plasma membrane proteins and extracellular material through the endosomal system, whereas macropinocytosis enables cells to internalise relatively large volumes of extracellular fluid and macromolecules that can subsequently be degraded to provide nutrients. In specialised cells, phagocytosis delivers microorganisms, cellular debris and other large particles to phagolysosomes, linking lysosomal degradation to innate immunity and antigen processing. These pathways are particularly relevant in cancer because they allow tumour cells to recover nutrients during metabolic stress, regulate the abundance of signalling receptors, remove damaged cellular components and modify interactions with the immune microenvironment.

In this review, we use the term lysosomal capacity to refer to the integrated ability of lysosomes to receive cargo, maintain luminal acidity, sustain hydrolase activity, preserve membrane integrity, export degradation products and coordinate nutrient‐sensing and stress‐adaptive signalling programmes.

Figure 1 provides the roadmap for this review. We first discuss lysosomal catabolism and metabolite transport in cancer, then examine how the lysosomal surface integrates nutrient signals, how membrane contact sites connect lysosomes to other organelles in cancer, and finally how lysosomes shape tumour immunity. Across these sections, we discuss tumour reliance on lysosomes and highlight where lysosomal states may create biomarkers or therapeutic vulnerabilities.

**Fig. 1:**
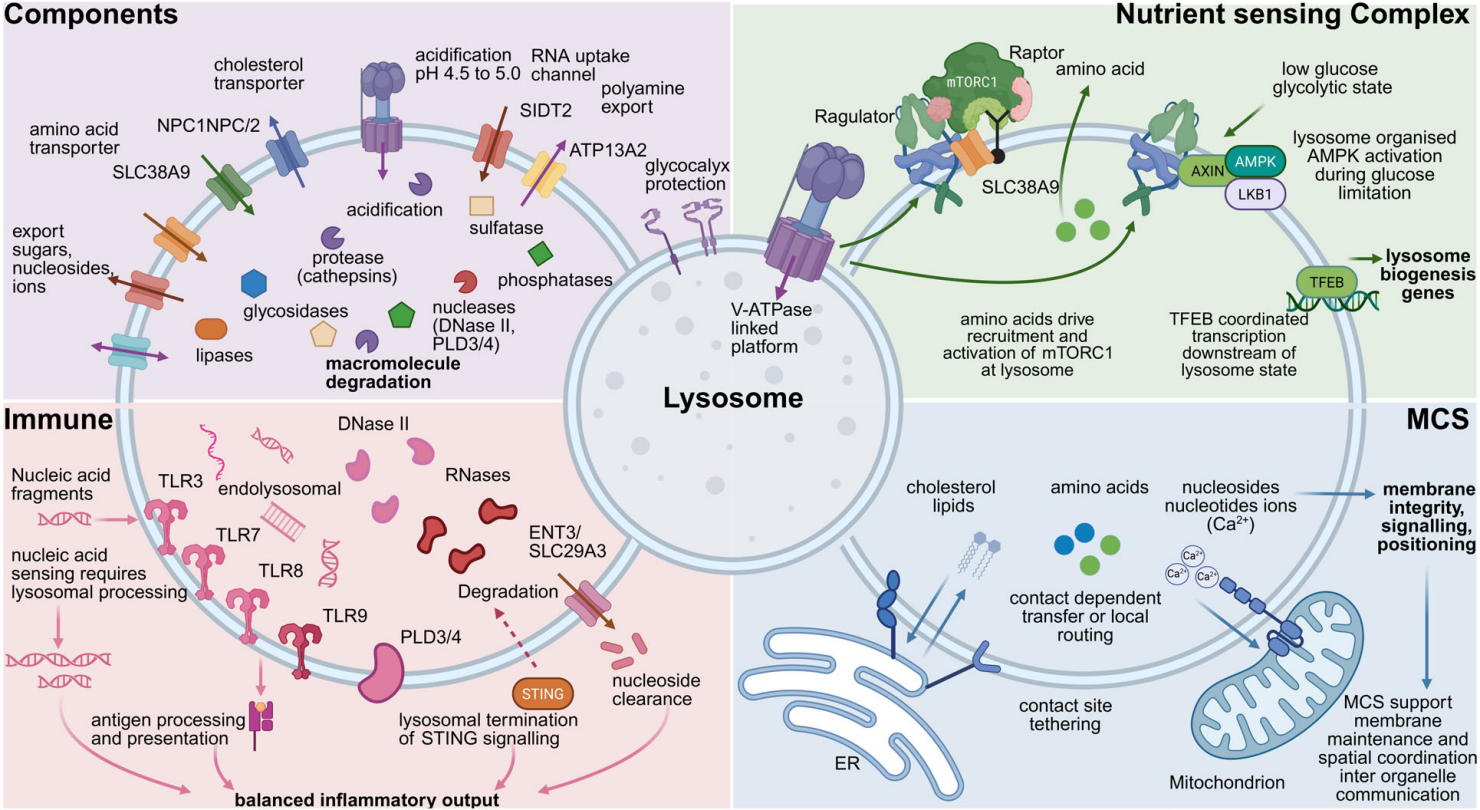
Summary roadmap of lysosome functions in cancer. The lysosome integrates four interconnected layers that shape tumour cell fitness and immune responses. Catabolic components include luminal hydrolases and specialised membrane systems such as the v‐ATPase, LAMP glycocalyx, and transporters that export amino acids, sugars, lipids, nucleosides, and ions following macromolecule degradation. Nutrient sensing occurs at the lysosomal surface, where Ragulator and v‐ATPase‐dependent assemblies recruit mTORC1 in response to amino acids, while lysosome‐organised AMPK activation coordinates energy stress responses during glucose limitation, with TFEB linking lysosome state to transcriptional programmes. Membrane contact sites (MCS) connect lysosomes with the ER and mitochondria to support lipid transfer, calcium coupling, membrane maintenance, signalling, and organelle positioning, and may enable contact‐dependent metabolite routing. Immunity is shaped through antigen processing and endolysosomal nucleic acid sensing by TLRs, balanced by nucleases and nucleoside transporters that clear ligands and by lysosome‐dependent termination of STING signalling to prevent sustained inflammation. Abbreviations: AMPK, AMP‐activated protein kinase; ER, endoplasmic reticulum; LAMP, lysosome‐associated membrane protein; mTORC1, mechanistic target of rapamycin complex 1; STING, stimulator of interferon genes; TFEB, transcription factor EB; TLR, Toll‐like receptor; v‐ATPase, vacuolar‐type H^+^‐ATPase.

## 2 Lysosomal catabolism and transport in cancer

Tumour expansion creates high nutrient demand, making lysosomal catabolism and nutrient recycling increasingly important. Figure 2 maps lysosome organisation, highlighting luminal hydrolases and membrane transporters that convert incoming cargo into recyclable nutrients.

**Fig. 2:**
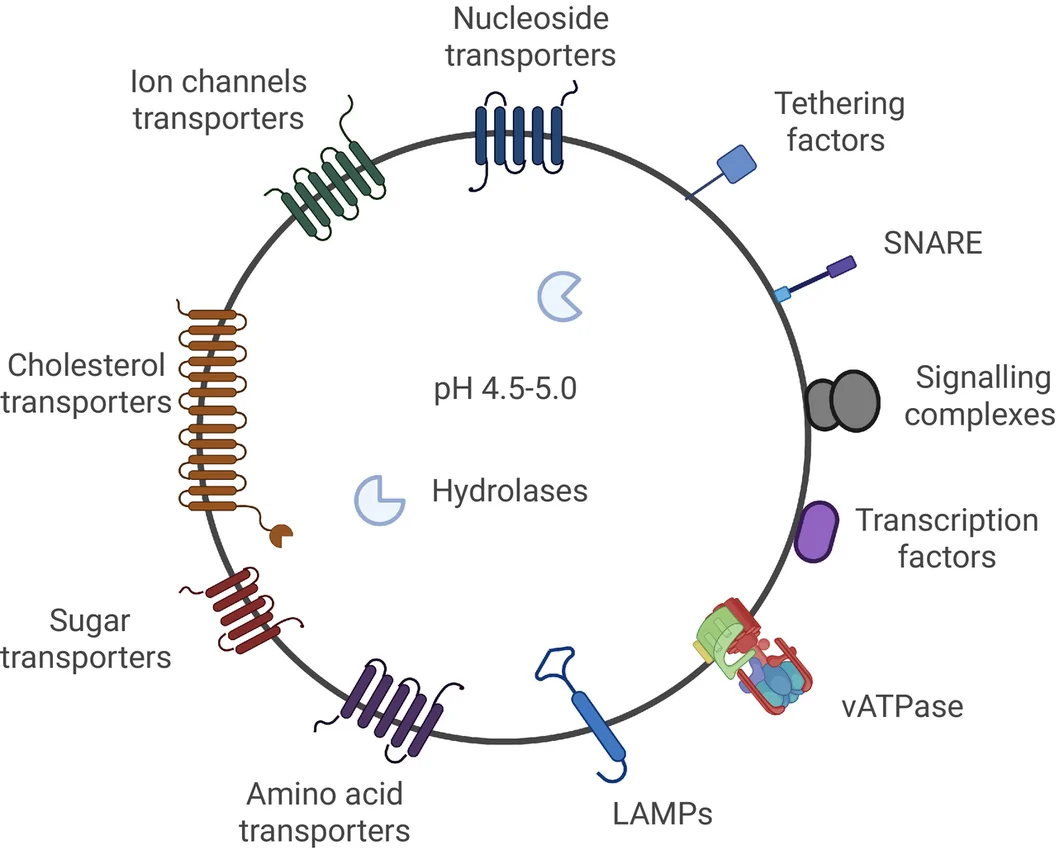
Lysosome properties and oncology‐relevant outputs. Lysosomes contain multiple effectors that support cellular homeostasis and, in cancer, nutrient scavenging and stress tolerance. Catabolism: luminal acidity is maintained at approximately pH 4.5–5.0 by the vacuolar‐type H^+^ ATPase, enabling acid hydrolases to degrade macromolecules. Transporters: cholesterol, sugars, amino acids, and nucleosides are transported alongside ions that maintain ionic balance and membrane potential. LAMP: highly glycosylated lysosome‐associated membrane proteins form a protective glycocalyx that shields the limiting membrane from luminal hydrolases. Nutrient sensing: lysosome biogenesis and function are transcriptionally coordinated by TFEB. In parallel, mTORC1 and AMPK localise to the lysosomal surface to sense nutrient availability and modulate catabolic programmes. Membrane fusion: SNARE proteins mediate fusion with endosomes and autophagosomes, while tethering factors support inter‐organelle contacts. Together, these features link lysosome state to tumour growth, metabolic flexibility, redox balance and therapy adaptation. Conceptually informed by Ballabio and Bonifacino (Nature Reviews Molecular Cell Biology, 2020). Abbreviations: AMPK, AMP‐activated protein kinase; LAMP, lysosome‐associated membrane protein; mTORC1, mechanistic target of rapamycin complex 1; SNARE, soluble NSF attachment protein receptor; TFEB, transcription factor EB; v‐ATPase, vacuolar‐type H^+^‐ATPase.

The lysosomal lumen is maintained at pH ~4.5–5.0, which is essential for hydrolase activity. v‐ATPase plays a key role in sustaining this acidic environment. v‐ATPase activity is frequently enhanced in cancer; increased v‐ATPase subunits expression has been reported in tumours, and their depletion suppresses tumour growth and metastatic progression [1, 2, 3, 4, 5, 6, 7].

Within the lysosomal lumen, more than 60 acid hydrolases have been identified. Table 1 lists proteases, nucleases, lipases and glycosidases that degrade diverse cargo, and several are upregulated or activated across tumour types to support tumour growth [8, 9, 10, 11, 12, 13, 14, 15, 16, 17].

**Table 1:** Lysosomal enzymes: Substrates, representative hydrolases and key functions.

Enzyme	Substrates	Representative gene	Function and ref
Proteases	Peptides and proteins, aminopeptidase substrates	CTSD, CTSB, CTSL, CTSS, PLBD1	Core proteolysis generating amino acids and peptides for export and recycling (Cathepsins) [254]. Lysosomal aminopeptidase activity supporting peptide processing and salvage (PLBD1) [45].
Glycosidases	Glycans (N‐linked and O‐linked), glycolipids, glycosaminoglycans	MAN2B1, HEXA, HEXB, NEU1, GLB1, GUSB, IDUA	Breakdown of carbohydrate‐rich cargo; impairment is strongly linked to storage phenotypes (MAN2B1, HEXA, HEXB, NEU1, GLB1, GUSB, IDUA) [255].
Lipases	Lipids and phospholipids, BMP (lysobisphosphatidic acid)	LIPA, PLA2G15	Lipid catabolism linking lysosomal membrane organisation with lipid storage (LIPA) [256]. BMP catabolism as part of lysosomal lipid handling (PLA2G15) [66].
Sulfatases	Sulfated glycosaminoglycans	ARSA or ARSB, IDS	Essential for glycosaminoglycan degradation; enzyme defects are frequent causes of lysosomal storage disorders (arylsulfatases, IDS) [255].
Nucleases	DNA, RNA	DNASE2, RNASET2, PLD3, PLD4	Lysosomal nucleic acid handling with implications for immune signalling (DNASE2) [257]. Endolysosomal single‐stranded nucleic acid degradation shaping inflammatory outputs (PLD3, PLD4) [69].
Phosphatases	Phosphomonoesters, nucleotides	ACP2	Final dephosphorylation steps during macromolecule breakdown (ACP2) [258].
Other hydrolases	Glycosaminoglycans, glycoproteins	GUSB, FUCA1	Complements glycosidases to complete complex carbohydrate processing (GUSB, FUCA1) [255].
Lipid synthase	BMP (lysobisphosphatidic acid)	PLD3, PLD4, CLN5	Links lysosomal membrane lipid organisation to degradative efficiency through BMP synthesis (PLD3, PLD4, CLN5) [55, 259].

The products of lysosomal hydrolysis are then exported to the cytosol by transporters for biosynthesis and signalling. Table 2 groups these systems into amino acid and peptide, sugar, ion, lipid and nucleic acid transport. Several are upregulated or functionally required in cancer, increasing lysosomal nutrient output [18, 19, 20, 21, 22, 23, 24, 25, 26, 27, 28, 29, 30].

**Table 2:** Lysosomal transport systems: substrates, representative transporters, and key functions.

Transporter	Main substrates	Representatives	Function and ref
Lipid	Cholesterol, glycerophosphodiesters (GPDs) and related lipids	NPC1, NPC2 and CLN3	Exports lysosomal cholesterol for cellular lipid metabolism (NPC1, NPC2) [83, 84]. Controls glycerophosphodiester clearance (CLN3) [88].
Sugar	Hexoses	SPNS1, GLUT8 (SLC2A8), SLC37A2, SLC45A1	Putative sugar transporter in Drosophila (SPNS1) [134]. Lysosome‐targeted glucose transporter with a lysosomal sorting motif (GLUT8/SLC2A8) [132]. Sugar transporter in osteoclast secretory lysosomes (SLC37A2) [260]. Sugar transporter in brain cells (SLC45A1) [130].
Amino acid and peptide	Amino acids, dipeptides	SLC38A9, PHT1 (SLC15A4), PHT2 (SLC15A3), MFSD1–GLMP heterodimer, MFSD12, CTNS	Lysosomal amino acid transporter and sensor coupling luminal amino acids to mTORC1 (SLC38A9) [20, 71, 90, 91]. Endolysosomal dipeptide transporters supporting immune signalling and metabolism (PHT1/SLC15A4, PHT2/SLC15A3) [93, 96]. Broad‐capacity dipeptide export under high proteolytic load (MFSD1–GLMP) [94, 95, 100]. Lysosomal cyst(e)ine importer (MFSD12) [19, 92] and exporter (CTNS) [261].
Ion	Potassium, protons, chloride; Iron, nitrogen‐containing bisphosphonates	TRPML1; TMEM175; PAC; SLC37A3–ATRAID; SLC7A11	Lysosomal Ca^2+^/Fe^2+^ channel (TRPML1) [119, 122]. Lysosomal K^+^ transporter (TMEM175) [114, 115]. Lysosomal chloride channel (PAC) [117]. Transport complex for nitrogen‐containing bisphosphonates (SLC37A3–ATRAID) [124]. Unconventional lysosomal H^+^ transporter (SLC7A11) [111, 112].
Nucleic acid	Nucleic acids (RNA), nucleic acid catabolites	SLC29A3 (ENT3); SIDT2	Lysosomal nucleoside transporter (SLC29A3/ENT3) [104]. Mediates RNA uptake into lysosomes (SIDT2) [108].
Other	Polyamines	ATP13A2	Lysosomal membrane transporter mediating polyamine export (ATP13A2) [126, 249].

The limiting membrane of the lysosome is protected from luminal hydrolases by lysosome‐associated membrane proteins (LAMPs) that form a highly glycosylated layer that protects. Increased LAMP expression has been reported to enhance lysosomal resilience and support tumour cell adaptation to stressful microenvironments [31, 32, 33]. Moreover, cancer cells can also increase hydrolase and transporter activity and v‐ATPase function to expand lysosomal capacity and convert autophagic and endocytic cargo into nutrients.

Below, we focus on recent findings in the hydrolases and lysosome transporters that expand lysosomal catabolism and may reveal context‐specific bottlenecks in nutrient salvage and therapy tolerance.

### 2.1 Lysosomal enzymes in tumour catabolism and progression

Classical lysosomal hydrolases underpin catabolic capacity by degrading proteins, glycans, lipids, and nucleic acids through well‐established enzyme groups including cathepsins, glycosidases, sulfatases, nucleases and phosphatases, generating reusable building blocks for export and recycling.

Recent studies have assigned new substrates or reactions to several lysosomal enzymes, broadening our view of lysosomal throughput. These studies focus on three themes: protein and peptide degradation, BMP‐dependent lipid remodelling and nucleic acid handling, revealing potential cancer targets.

Lysosomal protein and peptide degradation helps cancers meet nutrient demand for protein synthesis and biomass production. Lysosomal proteases, particularly cathepsins, degrade proteins and peptides into smaller peptides and amino acids for recycling and are established contributors to tumour progression. Elevated cathepsin B has been observed in several solid tumours and linked to therapy resistance, including chemotherapy [34, 35, 36]. Extracellular cathepsin B can remodel the extracellular matrix and tumour microenvironment to promote invasion, metastasis and chemotherapy [35, 37, 38, 39]. Cathepsin D exists in precursor, intermediate and mature forms, and its expression, processing and secretion have been linked to tumour progression, prognosis, therapy response and immune regulation [40, 41]. In colorectal cancer, cathepsin D‐mediated MHC class I degradation promotes invasion [42].

Recent work has also expanded the lysosomal protease repertoire and identified new candidate targets in cancer. PLBD1 (phospholipase B domain containing 1) was annotated as a phospholipase, although its activity was poorly defined [43, 44]. It has now been identified as the lysosomal leucine aminopeptidase LyLAP, which processes membrane‐derived proteins and peptides; PLBD1 loss causes lysosomal damage and pancreatic ductal adenocarcinoma cell death [45]. This identifies PLBD1 as a potential cancer immunotherapy target by regulating plasma membrane protein degradation and potentially influencing antigen presentation.

In addition to the modulation of proteases, BMP‐dependent remodelling of lipids is also important in the control of lysosome function. BMP, bis(monoacylglycero)phosphate, also known as lysobisphosphatidic acid (LBPA), is a major lipid component supporting lysosomal membrane organisation and degradation. Enriched in intraluminal membranes of late endosomes and lysosomes, its unusual stereochemistry promotes stability in the acidic lysosomal environment and resistance to acid hydrolysis. BMP remodelling is highly cancer‐relevant. Breast cancer cells can depend on BMP enrichment to resist lysosomal membrane permeabilisation [46]. BMP‐rich tumour‐derived extracellular vesicles have also been reported to increase immunogenicity and antitumour immunity [47, 48].

The lysosomal proteins PLD3 (phospholipase D3) and PLD4 (phospholipase D4) have also been found upregulated in oesophageal squamous cell, gastric and colorectal cancers [15, 49, 50, 51]. Although originally annotated as phospholipases, PLD3 and PLD4 have now been shown to synthesise BMP, linking lysosomal lipid composition to degradative efficiency and lipid‐storage phenotypes [52, 53, 54, 55]. Similarly, CLN5 (ceroid lipofuscinosis neuronal protein 5), a lysosomal protein associated with neuronal ceroid lipofuscinosis (Batten disease), has been proposed to function as a BMP synthase [56, 57, 58]. CLN5 deficiency causes lysosomal storage and impaired lysosomal homeostasis, leading to progressive neurodegeneration [59, 60, 61]. Importantly, CLN5 deficiency has also been reported to suppress glioblastoma cell growth [62].

Conversely, PLA2G15 (phospholipase A2 group XV), previously characterised as a lysosomal phospholipase, has been proposed to act as a BMP hydrolase, and targeting this enzyme has been explored in Niemann–Pick disease type C1 [56, 63, 64, 65, 66]. This suggests a potential control point for BMP turnover, although direct tumour evidence remains limited. Together, these findings identify BMP synthesis and turnover as a coordinated lipid‐remodelling axis; enzymes controlling BMP abundance may therefore influence both tumour survival and immune responses.

A third theme of lysosomal function relevant to cancer is lysosomal nucleic acid processing. Nucleoside and nucleic acid recycling may be particularly important in proliferating tumours because DNA replication and RNA synthesis impose high nucleotide demand. The lysosomal DNA nuclease DNASE2 mediates DNA breakdown and has been linked to glioblastoma and hepatocellular carcinoma progression [67, 68]. PLD3 and PLD4, discussed above as BMP synthases, also function as endolysosomal exonucleases [69]. This nuclease activity is also relevant to tumour immunity because degradation of internalised nucleic acids limits ligand availability to endolysosomal sensors, linking lysosomal catabolism to inflammatory signalling discussed in Section 4.

Together, lysosomal proteases, lipases and nucleases influence tumour growth, invasion, and immune responses while providing candidate biomarkers and tumour‐selective vulnerabilities.

### 2.2 Glucose and amino acid sensing

Distinct nutrient sensors, including amino acid sensors and glycolysis‐linked metabolites, converge on lysosomal signalling to tune mTORC1 activity and coordinate AMPK activation during glucose limitation. As such, lysosomal signalling integrates transported nutrients with cytosolic metabolic state.

For amino acids, SLC38A9 and cytosolic CASTOR1 link arginine sufficiency to mTORC1, while Sestrin2 and SAMTOR sense leucine and methionine [70, 71, 72, 73]. Glucose sensing is mediated by mTORC1 and AMPK at the lysosomal surface. mTORC1 responds through Rag‐dependent and glycolysis‐linked mechanisms involving hexokinase II, glucose‐6‐phosphate, dihydroxyacetone phosphate and glyceraldehyde‐3‐phosphate [74, 75, 76, 77], whereas AMPK is classically activated by low energy [78], but glucose starvation can activate AMPK without measurable changes in energy charge [79, 80, 81]. Under low glucose, fructose‐1,6‐bisphosphate/aldolase signalling and the v‐ATPase‐Ragulator‐AXIN‐LKB1 axis organise AMPK activation at lysosomes [79, 80, 81]. Thus, lysosomes integrate recycled nutrients with glycolytic state to shape tumour growth, survival and therapy response in nutrient‐variable microenvironments.

### 2.3 Lysosomal transporters in tumour metabolism and homeostasis

Cancer cells require continuous nutrient salvage to meet high metabolic demand. Following degradation, distinct lysosomal transporters release or exchange lipids, sugars, amino acids and peptides, ions, nucleosides and other metabolites, supporting growth, stress tolerance and therapy adaptation. Recent work broadens this transporter landscape from classical nutrient routes, including lipids, amino acids and peptides, and nucleic acids, to ion homeostasis and unconventional substrates.

#### 2.3.1 Lipid transporters

Lipids and lipid‐derived metabolites are in high demand for membrane synthesis during tumour proliferation and also support cancer progression and therapy resistance [82]. NPC1 and NPC2 (Niemann–Pick C1 and C2 proteins) mediate lysosomal cholesterol export [83, 84]. NPC1 inhibition has been reported to enhance chemotherapy responses in triple‐negative breast cancer [21]. CLN3 (ceroid lipofuscinosis neuronal protein 3) is a lysosomal membrane protein associated with neuronal ceroid lipofuscinosis (Batten disease) [85], which was found elevated in hepatocellular carcinoma, glioblastoma, neuroblastoma, prostate, ovarian, breast and colorectal cancers, and its loss can induce tumour cell apoptosis [24, 30, 86, 87]. Recent work identified CLN3 as a lysosomal glycerophosphodiester transporter [88]. This function provides a mechanistic basis for considering CLN3 dependence in highly proliferative tumours with high membrane lipid demand.

#### 2.3.2 Amino acid and peptide transporters

Lysosomal amino acid and peptide transport helps cancers meet nutrient demand for protein synthesis and biomass production. The transporter SLC38A9 (solute carrier family 38 member 9) was initially linked to sodium‐coupled amino acid transporters [89] and is now established as a lysosomal arginine transporter [20, 71, 90, 91]. SLC38A9‐mediated lysosomal amino acid efflux sustains pancreatic tumour growth [20]. MFSD12 (major facilitator superfamily domain‐containing 12) mediates lysosomal cyst(e)ine handling and promotes oxidative stress tolerance in cancer cells [19, 92].

Peptide export remains less understood. PHT1 (SLC15A4) and PHT2 (SLC15A3), previously poorly characterised lysosomal membrane proteins [93], are endolysosomal dipeptide transporters that support metabolism and immune signalling in glioblastoma [94, 95, 96]. MFSD1, another previously poorly characterised lysosomal protein [97, 98], forms a heterodimer with the glycosylated lysosomal membrane protein GLMP [99] to support broad‐capacity dipeptide export under high lysosomal proteolytic load [100]. Overall, these studies revealed that lysosomal amino acid and peptide export can support tumour growth [101].

#### 2.3.3 Nucleoside and nucleic acid transporters

Similarly, cancer cells depend on lysosome‐supported nucleotide pools to maintain precursors for DNA replication and RNA synthesis during tumour expansion [102]. Lysosomal nucleoside and nucleic acid transport helps proliferating tumour cells meet nucleotide demand. Equilibrative nucleoside transporters (ENTs; SLC29 family) regulate nucleoside transport [103]. ENT3 is a lysosomal nucleoside transporter [104] and has been reported to be upregulated and tumour‐promoting in hepatocellular carcinoma [105]. SIDT2 (SID1 transmembrane family member 2) mediates RNA and DNA uptake into lysosomes [106, 107, 108] and has been linked to gastrointestinal tumour development [26]. Together, these routes support the importance of lysosomal‐mediated nucleotide recycling in proliferating tumours.

#### 2.3.4 Ion channels

Beyond nutrient transport, lysosomal ion channels and transporters regulate cellular and lysosomal homeostasis, which becomes increasingly important as tumour cells increase lysosomal workload. For example, v‐ATPase is the principal driver of lysosomal acidification and additional potassium, chloride and proton channels contribute to pH and membrane potential control [109]. SLC7A11, known as a cystine/glutamate antiporter [110], also functions as a lysosomal H^+^ transporter regulating pH in several cancer cell lines [111, 112]. TMEM175 (transmembrane protein 175), initially reported as a lysosomal H^+^ transporter [113], also conducts K^+^ and supports membrane potential and pH stability [114, 115]. TMEM175 deletion in macrophages results in lysosomal permeabilization and impaired tumour growth [116]. The proton‐activated chloride channel PAC contributes to lysosomal chloride conductance and pH control [117], and PAC knockout impaired colorectal cancer cell growth [118]. Together, these findings suggest that tumours depend on ion transport to maintain lysosomal proton homeostasis and fitness under high workload.

Beyond pH control, lysosomal ion transport also regulates ferroptosis, lysosome biogenesis and drug responses through iron, Ca^2+^ and nitrogen‐containing bisphosphonate handling. TRPML1 (transient receptor potential mucolipin 1) functions as a lysosomal Fe^2+^ channel implicated in ferroptosis [119, 120]. Lysosomal iron is a key determinant of ferroptotic sensitivity in cancer; TRPML1 therefore provides a targetable vulnerability [121]. It also acts as a lysosomal Ca^2+^ channel controlling lysosomal signalling and biogenesis [122], and has been reported to promote breast cancer, H‐Ras‐driven cancer and melanoma [22, 23, 120, 123]. Interestingly, the SLC37A3 (solute carrier family 37 member 3)‐ATRAID (all‐trans retinoic acid‐induced differentiation factor) complex transports nitrogen‐containing bisphosphonates from lysosomes and thereby shapes anticancer drug responses [124]. This study links lysosomal transport directly to drug sensitivity. In addition, lysosomes also transport polyamines, metabolites that support nucleic acid organisation, translation and tumour cell growth. ATP13A2 (PARK9) is a lysosomal ATPase that exports polyamines and supports polyamine metabolism, tumour growth and therapy resistance through effects on nucleic acid organisation, translation and stress responses [125, 126, 127]. This broadens the lysosomal metabolite landscape and suggests polyamine export as a potential cancer dependency.

Together, these findings suggest that cancers tune lysosomal catabolism and transport to meet nutrient demand while maintaining ion homeostasis.

### 2.4 Questions and future directions

Lysosomal storage disorders can help reveal the functions of poorly characterised lysosomal genes, thereby uncovering catabolic pathways that may also create nutrient‐recycling dependencies in cancer. At the same time, reannotation of known lysosomal proteins continues to reveal unexpected functions. PLD3 and PLD4, for example, have been linked to both lipid remodelling and nucleic acid processing, whereas TMEM175 contributes to both proton and potassium transport. These findings highlight the need to consider multifunctionality when defining the roles of lysosomal enzymes and transporters.

An additional challenge is that lysosomal proteins can have tumour‐specific effects. Cathepsin D, for example, has tumour‐promoting roles in glioma and colorectal cancer, whereas high CTSD expression has been associated with better prognosis in neuroblastoma [128]. Defining the tissue, genetic and disease stage contexts that determine whether individual lysosomal proteins promote or restrain tumour development will therefore be important.

Major gaps also remain in lysosomal substrate transport. Sugar transport is particularly poorly defined compared with lipid and amino acid transport. SLC45A1 was recently identified as a lysosomal sugar transporter in brain cells, and SLC45A1‐mutant neurological disease shows lysosomal‐storage‐disorder‐like features [129, 130, 131]. Notably, SLC45A1 lies within a region frequently deleted in neuroblastoma and has been reported to exert tumour‐suppressive effects in glioblastoma [28]. GLUT8 has been proposed as a lysosomal sugar transporter based on the presence of a lysosomal sorting motif [132], but direct evidence that GLUT8 mediates hexose efflux from lysosomes is limited.

Species differences add further uncertainty. Spinster was initially described as a putative sugar transporter in Drosophila, whereas human SPNS1 has instead been implicated in lysosomal phospholipid transport [133, 134]. Future studies should therefore establish transporter localisation and substrate specificity directly in human cancer models rather than relying solely on annotations derived from other species or tissues.

Lysosomal capacity is also determined by the delivery and maintenance of lysosomal components, not only by their expression. LYSET (lysosomal enzyme trafficking factor) links mannose‐6‐phosphate‐dependent enzyme trafficking to lysosomal proteome stability [135], illustrating how changes in biosynthetic delivery can reshape lysosomal function. Whether tumours alter such trafficking pathways to expand or maintain lysosomal capacity remains an important question.

These mechanistic uncertainties have direct therapeutic implications. Autophagy‐dependent lysosomal degradation can suppress tumour initiation, and loss of Beclin 1 or core autophagy genes, including ATG5, ATG7 and FIP200, promotes tumour formation in several settings [136, 137, 138, 139]. Lysosome‐targeted therapies may therefore need to account for tumour type and disease stage. Although chloroquine and hydroxychloroquine have been used to inhibit lysosomal degradation in cancer [140, 141, 142], broad lysosomal inhibition can also affect normal cells. More selective targeting of tumour‐dependent lysosomal enzymes, transporters or trafficking pathways may provide greater therapeutic specificity, while their expression or activity could serve as biomarkers of lysosomal dependence.

## 3 Lysosomes as nutrient‐sensing hubs

Lysosomes are not merely endpoints of degradation. Cancer cells rely on degradation, which feeds interconnected networks that adjust to context and demand. As shown in Fig. 2, the lysosomal surface is a spatial signalling platform enriched in regulatory complexes. Rag‐ and v‐ATPase‐dependent assemblies recruit and activate mTORC1 (mechanistic target of rapamycin complex 1), while related scaffolds organise AMPK activation to coordinate anabolic growth with energy stress. By coupling nutrient generation, sensing and signalling, lysosomes help tumour cells switch between biosynthesis and conservation in nutrient‐limited microenvironments.

mTORC1 is a central regulator of nutrient sensing and protein synthesis [143, 144]. Amino acids drive mTORC1 relocalisation to lysosomes through Rag GTPases and the v‐ATPase, while structural work has clarified docking of the active complex at the lysosomal membrane [145, 146, 147, 148, 149]. Upstream regulators including KICSTOR, GATOR1 and SAMTOR translate nutrient inputs into Rag‐state control [150]. This spatial control supports growth when nutrients are available while enabling rapid adaptation to intermittent starvation, shaping proliferation, autophagy and sensitivity to metabolic therapies.

In parallel, AMPK is increasingly understood as a lysosome‐organised stress response rather than a purely diffuse cytosolic sensor: under low glucose, pathways involving V‐ATPase and Ragulator recruit AXIN and LKB1 to the lysosomal surface, enabling efficient AMPK activation and coordinated suppression of anabolic signalling [81]. Together, these findings indicate that the lysosome is a privileged signalling location which couples nutrient generation, sensing and downstream execution, allowing cells and tumours to rapidly tune growth and autophagy in response to fluctuating supply.

### 3.1 Lipid and nucleotide sensing

Compared with amino acid and glucose sensing, lysosome‐linked lipid and nucleotide sensing is less well defined. Lysosomes integrate sterol status with nutrient signalling and positioning, processes that are important in tumours with sustained demands for membrane synthesis, lipid remodelling and nucleotide turnover.

ORP1L (oxysterol‐binding protein‐related protein 1‐like) and its partners VAP‐A and VAP‐B connect cholesterol status at late endosomes and lysosomes to NPC1‐dependent sterol export and to microtubule‐driven lysosome positioning [151], suggesting a spatial layer in which lipid state influences where lysosomes sit and therefore which signalling interactions they can form. The interaction between the cholesterol transporter NPC1 and amino acid transporter SLC38A9 provides a more direct route to mTORC1, indicating that a classic amino acid sensor module can also relay cholesterol cues [152]. This supports a broader model in which the lysosomal surface integrates orthogonal nutrient classes, and in cancer, where sterol demand and membrane remodelling are high, sterol‐linked tuning of mTORC1 may shape both growth and sensitivity to stress.

Purine sensing is also linked to lysosomal mTORC1 suggesting that nucleosides and related metabolites can contribute to nutrient‐sufficiency signalling [153]. Endolysosomal TLR7 (Toll‐like receptor 7) sensing of nuclease‐generated RNA fragments illustrates a related principle: lysosomal degradation can generate signalling ligands rather than simply terminate cargo [154]. In tumours, where nutrient recycling, nucleic acid turnover and inflammation coexist, these observations position lysosomes as hubs integrating metabolic and immune inputs.

### 3.2 Questions and future directions

Progress over the past decade has established the lysosomal surface as a central nutrient‐sensing platform. mTORC1 and AMPK provide the clearest examples of how lysosome‐localised complexes integrate nutrient availability with cell growth, autophagy and metabolic rewiring. However, the prevailing framework remains heavily centred on these two signalling axes, and this emphasis may obscure additional lysosome‐based sensing systems that operate in parallel, upstream, or in distinct contexts.

One important direction is to determine whether lysosomes sense metabolic states beyond nutrient abundance. Nutrients also support cofactors and redox metabolism, including NADPH, NADH and FAD dependent processes. Whether lysosomes directly or indirectly detect nutrient‐derived redox states and translate them into adaptive responses remains poorly understood. Such mechanisms could be particularly relevant in cancer, where redox control influences responses to radiotherapy, chemotherapy and oxidative‐stress‐induced cell death.

Lysosomal catabolism may also generate metabolites that require dedicated sensing mechanisms. For example, degradation of nucleic acids produces nucleosides that can be salvaged for nucleotide synthesis, yet how cells sense lysosome‐derived nucleoside availability remains unclear. This is potentially linked to mitochondrial nucleotide homeostasis, since mitochondria rely on nucleotide import for mtDNA and mtRNA synthesis due to limited *de novo* biosynthetic capacity. Whether lysosomal nucleoside or nucleotide sensing interfaces with mitochondrial nucleotide import, and how this coordination is achieved, remains unknown. Defining this link could clarify how tumour cells maintain mitochondrial transcription and replication during stress, which may shape persistence under treatment.

Beyond metabolic sensing, lysosomes may also act as stress‐responsive signalling platforms. Partial lysosomal membrane permeabilisation or controlled release of luminal ions and enzymes can activate inflammatory, cell death and adaptive responses. Determining how cells distinguish productive lysosomal signalling from damaging lysosomal rupture will be important for understanding when lysosomal stress promotes tumour survival, immune activation, or cell death.

Together, these questions extend the concept of the lysosome from a nutrient‐sensing platform to a broader metabolic and stress‐sensing organelle. Defining these additional sensing mechanisms, their tissue specificity and their interaction with immune and metabolic pathways may reveal new lysosome‐centred vulnerabilities in cancer.

## 4 Lysosomal membrane contact sites

As shown in Fig. 2, the lysosomal surface contains tethering factors and SNAREs (soluble NSF attachment protein receptors) that support membrane fusion and inter‐organelle communication. Lysosome membrane contact sites (MCSs) therefore place lysosomes within an inter‐organelle network rather than as isolated degradative units. In cancer cells, contact‐dependent communication can coordinate nutrient allocation, stress responses and organelle quality control under fluctuating nutrient availability and treatment pressure. These sites extend lysosomal function beyond bulk catabolite export by enabling spatially organised exchange with partner organelles. Here, we highlight cancer‐relevant contact site mechanisms and the gaps that must be resolved before they can be exploited therapeutically.

### 4.1 ER–lysosome contacts: Lipid, calcium and positioning

The ER is the most extensively studied organelle partner for lysosome membrane contact sites. ER lysosome MCSs act as multifunctional control hubs that integrate cholesterol and lipid handling with lysosome positioning, stress‐responsive membrane repair and calcium‐related signalling, and these coordinated outputs may support cancer cell survival under nutrient limitation and membrane stress.

Across the ORP1L–VAP and ORP5–NPC1 axes, ER–lysosome coupling senses lysosomal cholesterol status and converts it into coordinated changes in contact formation, lipid transfer and organelle distribution, including fusion control [151, 155, 156]. Under stress, contact sites appear to be repurposed towards lysosomal membrane protection, with ORP1L recruitment and VPS13C engagement supporting protective lipid transfer to damaged lysosomes [157, 158, 159, 160]. In tumours, these mechanisms are well placed to support metabolic plasticity because cholesterol and membrane lipid availability constrain endolysosomal trafficking, growth factor receptor turnover and mTORC1 signalling.

The expanding tether set, including TMEM55B, TEX2, STRAD3 and PDZD8, further indicates that contact composition is context‐ and stress‐dependent [161, 162, 163]. Altered ER–lysosome coupling has also been linked to disrupted calcium homeostasis through APP C‐terminal fragment‐mediated tethering and DRAM1–STIM1 interactions [164, 165]. In addition, a LIMP2–LC3B–TBC1D15 tether promotes calcium transfer and ER‐stress‐induced apoptosis, suggesting that manipulating organelle contacts may provide an antitumour strategy [166].

Contacts also influence organelle dynamics: VAP–STARD3‐dependent coupling can alter lysosome mobility through actin regulators, while lysosome anchorage shapes ER tubule distribution and elongation [167, 168]. This spatial organisation is cancer‐relevant because perinuclear and peripheral lysosome pools differ in mTORC1 activity, exocytosis and invasive behaviour.

Overall, ER–lysosome contacts integrate lipid transfer, membrane protection, calcium signalling and positioning. They are attractive tumour stress‐adaptation nodes, but many components are essential housekeeping factors; identifying cancer‐selective dependencies will therefore require context‐specific perturbations and quantitative flux and signalling readouts.

### 4.2 Mitochondria–lysosome contacts: Fission and metabolite exchange

Mitochondria and lysosomes are functionally interdependent: dysfunction in one can propagate to the other in neurodegenerative and lysosomal storage disorders, and loss of NPC1/2 can impair both lysosomal and mitochondrial function [169]. Mitochondria–lysosome contacts (MLCs) therefore provide a useful framework for understanding the molecular basis of this inter‐organelle crosstalk.

MLCs form spatial interfaces that coordinate lysosomal acidification, mitochondrial fission, lipid and ion exchange, redox homeostasis and organelle positioning. Importantly, this coupling is also cancer‐relevant, as MLCs support tumour fitness by maintaining both lysosomal and mitochondrial function [170, 171].

VDACs (voltage‐dependent anion channels) are abundant outer mitochondrial‐membrane channels controlling metabolite and ion exchange, bioenergetics and apoptosis. Their pro‐survival metabolic functions and pro‐apoptotic activity give them context‐dependent roles in cancer [172, 173, 174]. Accordingly, both pharmacological closure and opening of VDAC1 have produced antitumour effects in lung and breast cancer models by disrupting mitochondrial metabolism or inducing ROS‐dependent cell death, respectively [173].

At mitochondria–lysosome contacts, VDAC1 links local calcium transfer to broader mitochondrial metabolic and death decisions. TRPML1‐dependent lysosomal calcium release can couple to mitochondrial VDAC1, enabling local calcium transfer that shapes oxidative stress responses and selective mitochondrial remodelling [175, 176, 177]. These findings suggest an additional route through which VDAC1 may support tumour adaptation, by coupling mitochondria–lysosome contact site signalling to calcium and stress responses.

Recent work shows that mitochondria can support lysosomal acidification through membrane contacts, and experimentally increasing mitochondria–lysosome proximity enhances acidification and cargo digestion [178, 179]. Conversely, lysosomes help organise mitochondrial fission, with Rab7, a lysosomal small GTPase, and TBC1D15, a Rab7 GTPase‐activating protein, marking fission sites within multi‐organelle contact architectures [180, 181]. In addition, a LAMP1–GDAP1 contact axis has also been reported to couple lysosomal morphology with mitochondrial network organisation and glutathione homeostasis [182], and additional studies link lysosome‐mediated inflammatory cell death programmes to cGAS‐STING through a mitochondria–lysosome axis that connects mitochondrial DNA release to pyroptotic wiring [183].

It is considered that nutrient transfer occurs between mitochondria and lysosomes, but the concept is relatively new and requires further investigation. In short, Parkin stabilisation of Rab7 has been linked to amino acid balancing, although how amino acids move from lysosomes to mitochondria remains unclear [184]. Other disease and metabolic contexts include high‐glucose TM4SF5‐dependent contacts that support NPC1‐mediated cholesterol delivery to mitochondria and a proposed BDH2–DHBA axis for Fe^2+^ transfer that may influence ferroptosis sensitivity [170, 185, 186]. In tumours, these observations raise the possibility that contact sites tune ferroptosis thresholds through iron handling, mitochondrial lipid composition and redox state, but direct flux measurements are still needed.

### 4.3 Expanded lysosomal contact networks

Beyond the ER and mitochondria, lysosomes interact with additional cellular systems, including translation machinery, peroxisomes and the Golgi, extending their role in spatial proteostasis, lipid trafficking and nutrient‐responsive signalling.

Lysosome‐proximal translation illustrates how lysosomes can create local signalling and biosynthetic microenvironments. Lysosomes can act as mobile platforms for RNA granules and spatially restricted protein synthesis, processes regulated by Annexin A11, TRPML‐dependent calcium release, PI(3,5)P2 and BORC‐dependent positioning [187, 188, 189, 190].

ER‐associated lysosomes promoted by LNPK have also been linked to enhanced secretory pathway translation, local amino acid transport and increased transfer RNA synthesis, suggesting that lysosomal nutrient handling can be spatially coupled to secretome production [191].

Lysosomes also form contacts with peroxisomes and the Golgi involved in lipid and growth signalling. Lysosome–peroxisome contacts support NPC1‐dependent cholesterol export via SYT7 and PI(4,5)P2 [192], while Golgi–lysosome contacts have been observed during starvation and linked to mTORC1 regulation through RAB34, RILP, folliculin and Rheb [193, 194].

Together, these findings suggest that lysosome contact networks provide a spatial strategy for efficiency and prioritisation, bringing together nutrient release, translation, lipid redistribution, and mTOR‐related signalling to favour rapid adaptive programmes. This molecular basis raises the central question of whether tumours create stable, targetable contact site architectures that sustain growth and survival under microenvironmental stress.

### 4.4 Questions and future directions

Lysosomes are numerous and typically small, features that support their highly dynamic behaviour. Traditionally, this spatial advantage has been viewed mainly as a way to maximise lysosomal efficiency for degradation. An important open question is whether lysosome‐derived nutrient release occurs primarily as bulk export into the cytosol, or whether it can be spatially organised, with metabolites preferentially delivered to specific organelles or metabolic pathways. This distinction is especially relevant in tumours, where local metabolic constraints and heterogeneous nutrient access may favour spatially restricted nutrient routing to sustain growth and therapy resistance.

This possibility is increasingly plausible because lysosomes form extensive MCSs with other organelles and act as nutrient‐sensing platforms. Individual lysosomes may differ in nutrient status depending on local cargo and location. Inside‐out sensing modules such as mTORC1 could, in principle, label lysosomes with distinct signalling states, which might then influence lysosome positioning, contact site formation and spatial routing of nutrient export. Establishing this model would connect lysosome heterogeneity directly to tumour metabolic plasticity, but the mechanistic links between sensing, positioning and transfer remain unclear.

Several studies support the idea that direct cargo transfer can occur at lysosomal contact sites. Cholesterol exchange between the endoplasmic reticulum (ER) and lysosomes has been reported under different conditions, and lysosomes can also support directional cholesterol export to peroxisomes. Calcium transfer from lysosomes to mitochondria has been described at VDAC‐dependent contact sites. Lysosome contacts with lipid droplets have also been implicated in fatty acid transfer and utilisation [195]. For oncology, a priority is to define which of these transfers are rate‐limiting in tumour settings, and whether they become more important under therapy‐induced stress.

Two studies suggest that amino acid export can be spatially organised. Lysosome‐ribosome‐ER contacts can facilitate local amino acid supply for tRNA charging and secretory protein synthesis, while Parkin‐mediated mitochondria–lysosome contacts have been reported to promote lysosomal amino acid export to mitochondria [191]. These findings raise a key question: can lysosomes deliver amino acids and nucleosides directly to mitochondria, and does this sustain tumour mitochondrial function during nutrient limitation?

This idea is attractive from a metabolic perspective. Mitochondria lack several *de novo* nucleotide biosynthetic steps and therefore rely, at least in part, on imported nucleosides and nucleotides. It is therefore worth asking whether lysosomes can deliver nucleotides or nucleosides to mitochondria under specific stresses or nutrient states, and whether lysosomal nutrient‐sensing platforms actively regulate these contact‐dependent fluxes. At present, how sensing machinery, such as mTORC1, coordinates contact site dynamics and metabolite routing remains underexplored.

A related possibility is that lysosome proximity creates local metabolic microdomains. Growth factor stimulation can drive endocytic vesicles carrying glycolytic enzymes towards mitochondria [196], suggesting that vesicle trafficking can spatially couple carbohydrate processing to mitochondrial metabolism. Analogously, lysosome positioning might shape local hexose availability by concentrating sugar transporters, glycolytic enzymes or glycosylation capacity near lysosome‐mitochondria or lysosome‐ER contacts. This would directly link lysosomal positioning to tumour metabolic compartmentalisation.

## 5 Lysosomes in tumour immunity

### 5.1 Lysosomes in innate and adaptive antitumour immunity

Beyond their intracellular degradative and signalling functions, lysosomes shape communication between tumour cells and the immune system by controlling cargo clearance, antigen processing and inflammatory output. As summarised in Fig. 3, these functions span innate phagocytic clearance, antigen presentation to CD^4+^ and CD8+ T cells, B‐cell antigen processing, and the lysosome‐related secretory pathway used by cytotoxic lymphocytes. In tumours, this places lysosomes at a critical interface between the amount and quality of tumour‐derived material entering immune cells and the strength of the resulting antitumour response.

**Fig. 3:**
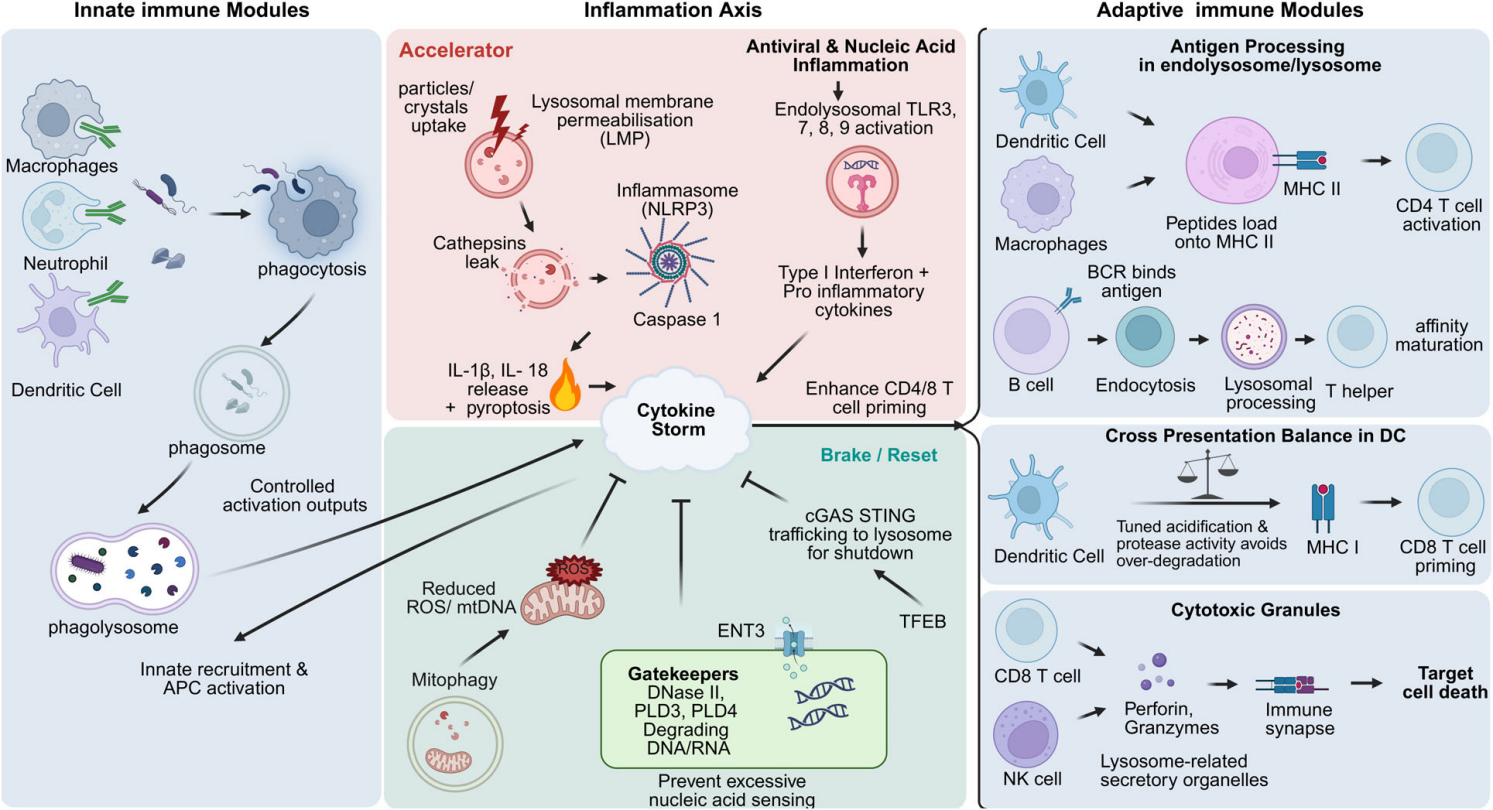
Lysosomal control of tumour immunity and inflammation. Lysosomes shape tumour immunity through three connected modules. Innate immune clearance: macrophages, dendritic cells and neutrophils engulf microbes, particles, crystals, dying cells and tumour‐derived debris; fusion with lysosomes generates phagolysosomes for cargo degradation and processing. Inflammatory axis: ‘Cytokine storm’ refers here to the combined local inflammatory cytokine milieu generated by pathways including NLRP3 activation and endolysosomal TLR signalling [262, 263, 264]. Lysosomal stress, membrane permeabilisation or endolysosomal TLR activation can promote cathepsin release, inflammasome activation, IL‐1β/IL‐18 production, type I interferon signalling and local cytokine output. Conversely, lysosomes can brake inflammation by degrading nucleic acid ligands through DNase II, PLD3 and PLD4, exporting nucleosides through ENT3 and terminating cGAS–STING signalling through lysosome‐dependent trafficking and degradation. Adaptive immune axis: lysosomal proteolysis supports MHC class II processing for CD^4+^ T‐cell activation, contributes to dendritic‐cell cross‐presentation that primes CD^8+^ T cells and enables B‐cell antigen processing. In effector lymphocytes, lysosome‐related cytotoxic granules containing perforin and granzymes mediate tumour cell killing. Together, these pathways place lysosomes at a checkpoint between tumour‐derived cargo uptake, inflammatory tone, antigen presentation and antitumour cytotoxicity. Abbreviations: cGAS, cyclic GMP–AMP synthase; DNase II, deoxyribonuclease II; ENT3, equilibrative nucleoside transporter 3; IL, interleukin; MHC, major histocompatibility complex; NLRP3, NLR family pyrin domain‐containing 3; PLD3, phospholipase D3; PLD4, phospholipase D4; STING, stimulator of interferon genes; TLR, Toll‐like receptor.

In innate immunity, macrophages, neutrophils and dendritic cells engulf microbes, cellular debris, particles and crystals by phagocytosis, after which phagosomes fuse with lysosomes to form phagolysosomes (Fig. 3, left). In tumours, macrophages and dendritic cells similarly ingest dying tumour cells and tumour‐derived proteins and nucleic acids. Lysosomal proteolysis generates peptides for MHC class II loading in professional antigen‐presenting cells, promoting CD^4+^ T‐cell activation [197, 198, 199] (Fig. 3, upper right). This pathway is particularly relevant after therapy‐induced tumour cell death when extracellular tumour material is abundant.

MHC class I presentation is essential for recognition of tumour cells by cytotoxic CD8+ T cells. In most nucleated cells, cytosolic proteins are degraded by the proteasome, and the resulting peptides are loaded onto MHC class I molecules in the endoplasmic reticulum before peptide–MHC class I complexes are transported to the cell surface. This constitutive pathway allows immune surveillance of the intracellular proteome and provides an important route through which transformed cells can be recognised by cytotoxic T cells.

Tumour cells can evade this surveillance by reducing surface MHC class I. Autophagy‐dependent lysosomal degradation of MHC class I has been reported in pancreatic ductal adenocarcinoma and hepatocellular carcinoma [200, 201], reducing cytotoxic T‐cell recognition and promoting immune evasion. Similarly, in colorectal cancer, cathepsin D promotes immune evasion by mediating MHC class I degradation, thereby reducing antigen presentation and CD8+ T‐cell activation [42].

Lysosomal antigen processing is also important in B cells. Antigen captured by the B‐cell receptor is internalised and delivered to endolysosomal compartments, where peptides are generated for MHC class II presentation to CD^4+^ T cells. Interaction with T follicular helper cells then supports B‐cell activation and affinity maturation. At the effector end of the adaptive response, CD^8+^ T cells and natural killer cells depend on specialised lysosome‐related secretory granules containing perforin and granzymes. Polarised release of these granules at the immune synapse provides a direct lysosome‐linked mechanism for killing tumour cells (Fig. 3, lower right).

A further layer of tumour immune escape arises through immune‐checkpoint pathways. Programmed death‐ligand 1 (PD‐L1), expressed by many tumour and immune cells, binds programmed cell death protein 1 (PD‐1) on activated T cells and suppresses T‐cell receptor signalling and effector function. This interaction provides the biological basis for therapeutic blockade of the PD‐1/PD‐L1 axis. Importantly, cell‐surface PD‐L1 abundance is itself regulated by intracellular trafficking, recycling and lysosomal degradation, directly linking lysosomes to immune‐checkpoint control [202, 203, 204, 205, 206].

Collectively, lysosomes determine whether tumour‐derived cargo is cleared, converted into antigenic peptides, presented to T cells or used to support antibody responses. Because tumour antigens are often limiting and heterogeneous, lysosomal control of antigen processing and receptor turnover may become a bottleneck for T‐cell priming and responses to antigen‐directed or checkpoint‐based immunotherapies.

### 5.2 Lysosomes as gatekeepers of inflammatory signalling

Lysosomes are immune decision hubs that coordinate initiation, amplification and termination of inflammatory signalling. As illustrated by the middle ‘accelerator’ and ‘brake/reset’ modules in Fig. 3, lysosomal compartments can generate inflammatory signals through nucleic acid sensing, membrane damage and inflammasome activation while clearing ligands and signalling proteins to prevent persistent activation. This balance is particularly important in cancer because tumours and therapies continuously generate damaged organelles, dying‐cell debris and nucleic acids that can drive either productive immunity or tumour‐promoting inflammation.

#### 5.2.1 Gatekeepers of nucleic acid inflammation

Lysosomes are key nucleic‐acid‐sensing organelles that enable controlled recognition of extracellular and intracellular nucleic acids through Toll‐like receptors (TLRs). Endolysosomes provide the spatial platform where nucleic‐acid‐sensing TLR3, TLR7, TLR8 and TLR9 are localised [207, 208, 209, 210, 211]. These receptors detect double‐stranded RNA [212], single‐stranded RNA [213, 214, 215], unmethylated CpG‐rich DNA [216, 217, 218]. This results in activation of NF‐κB and interferon‐regulatory factors to drive inflammatory cytokine and type I interferon production.

Lysosomes tightly coordinate these inflammatory responses by balancing nucleic acid sensing with TLR ligand degradation and clearance. Research evidence supports a useful duality: endolysosomes are the sites of nucleic acid sensing, yet they must also clear nucleic acids to avoid self‐directed activation. Lysosomal nucleases can regulate TLR responses by either generating or degrading receptor ligands. Lysosomal DNase II generates DNA fragments that can contribute to TLR9 activation [219, 220], whereas lysosomal exonucleases PLD3 and PLD4 negatively regulate TLR7 and TLR9 by degrading their ligands, with loss of these enzymes causing interferonopathy‐like phenotypes [15, 69, 219, 221].

Lysosomal nucleic acid transport can also adjust immune tone, defined here as the baseline level and character of immune and inflammatory activity within a tissue. ENT3 prevents nucleoside accumulation; its loss causes aberrant TLR7/8 activation [104, 222], and interferon can upregulate ENT3‐related transport programmes [223, 224].

These studies revealed that inflammatory activation can arise not only from excessive sensing, but also from failed clearance and metabolite accumulation following therapy‐induced debris influx. In tumours, this gatekeeping may determine whether nucleic‐acid‐rich debris generated by radiotherapy or genotoxic chemotherapy drives productive immunity or persistent dysfunctional inflammation.

#### 5.2.2 Lysosomal termination of cGAS–STING signalling

Lysosomes also regulate the dsDNA‐sensing cGAS‐STING pathway. A central advance is the recognition that lysosomes terminate STING signalling; failure of this shutdown can sustain tonic type I interferon states with major implications for tumour immunity and autoimmunity. Loss of factors required for STING trafficking to lysosomes can lock cells into persistent interferon output [225], while ESCRT‐dependent processes are required for lysosomal termination of STING signalling [226, 227].

The transcription factors TFEB/TFE3 couple lysosome biogenesis and function to immune activation programmes, linking cellular clearance to inflammatory gene expression and immune metabolism [228, 229]. Recent studies show TFEB/TFE3 participate in feedback control downstream of STING and can dampen type I interferon outputs [230, 231]. Conversely, cGAS–STING activation can activate TFEB and promote lysosome biogenesis, reinforcing autophagy‐related clearance capacity [232, 233].

Excessive lysosomal acidification in haematopoietic stem cells can impair mitochondrial DNA clearance and promote cGAS–STING activation [234], linking lysosomes to mitochondrial quality control and chronic inflammation. These studies indicated controlled STING activation may enhance antitumour immunity, whereas defective lysosomal termination or maladaptive feedback can sustain chronic interferon states with context‐dependent effects on tumour progression and immune composition.

#### 5.2.3 Lysosomal stress and inflammatory signalling

Loss of lysosomal integrity converts lysosomes from disposal compartments into danger amplifiers that trigger inflammasomes, inflammatory cell death and metal‐linked cytokine control. In cancer, poorly degradable particles, crystals, protein aggregates and drug‐induced lysosomal damage can affect tumour‐associated macrophages, dendritic cells and tumour cells, reshaping the tumour microenvironment.

Lysosomal stress responses can amplify inflammation, particularly when membrane integrity is compromised [235, 236]. Poorly degradable particles and lysosomal destabilisation can promote inflammasome activation, with lysosomal proteases such as cathepsins implicated in NLRP3‐related pathways [237, 238]. When lysosomal membranes are disrupted, exposed luminal glycans recruit galectins that act as damage sensors and can amplify inflammasome‐related responses, including non‐canonical inflammasome pathways during infection [239, 240].

Lysosomal membrane permeabilisation releases cathepsins and can activate NLRP3 and pyroptosis [35, 130, 241]. In cancer, pyroptosis may convert immunologically cold tumours into inflamed lesions by releasing cytokines and danger signals, potentially improving checkpoint blockade responses. This has motivated lysosome‐directed drug‐delivery strategies. For example, nanoscale assemblies formed within lysosomes can trigger membrane permeabilisation, cathepsin release and pyroptosis, enhancing antitumour immunity in preclinical models [242]. These observations support a context‐dependent model in which lysosomal stress can either enhance antitumour immunity through immunogenic cell death or promote tumour progression through chronic inflammation and matrix remodelling.

Beyond membrane rupture, lysosomal metal handling can tune inflammatory tone: TRPML1‐dependent Fe^2+^ release has been linked to transcriptional control of IL‐1β in macrophages [243], suggesting that lysosomal iron release can shape cytokine and innate immune programmes. Thus, lysosomal damage can amplify inflammation in the tumour microenvironment, either supporting antitumour immunity or promoting chronic inflammation and tissue remodelling depending on context.

#### 5.2.4 Lysosomal signalling and immunometabolism

Lysosomes organise nutrient sensing and couple catabolic state to immune‐cell transcriptional and metabolic programmes through mTORC1 signalling. Lysosomal mTORC1 integrates amino acid and growth factor inputs to regulate immune‐cell growth, differentiation and inflammatory tone [244]. This is particularly relevant to the tumour microenvironment, where heterogeneous nutrient availability can therefore alter the metabolic fitness of infiltrating lymphocytes and myeloid cells.

Lysosomes also regulate lymphocyte fate. NKG7‐mediated restraint of lysosome‐linked mTORC1 tunes CD^8+^ T‐cell effector programmes [245], while loss of folliculin impairs regulatory T‐cell function in a TFEB‐dependent manner [246]. Lysosomal signalling can therefore shape both cytotoxic antitumour responses and immunosuppressive cell states.

### 5.3 Questions and future directions

Lysosomes act as immune decision hubs that control antigen processing, nucleic acid sensing, metabolic rewiring and inflammatory termination during cancer immune response. Several questions remain.

For antigen processing, dendritic cells also cross‐present tumour antigens on MHC class I to prime CD8+ T cells (Fig. 3, middle right). Internalised antigens can be transferred from endosomes to the cytosol for proteasomal processing before MHC class I loading. However, whether autophagy‐dependent lysosome regulation of MHC class I turnover similarly influences dendritic‐cell cross‐presentation remains unresolved.

For nucleic acid sensing, the major question is how lysosomal nucleic acid balance operates under tumour‐relevant conditions. Endolysosomes activate nucleic acid sensors but must also destroy ligands to prevent sustained self‐directed signalling. Therapy‐induced cell death and chronic stress can flood tumours with nucleic‐acid‐rich debris. Defining how fragment generation, ligand destruction and metabolite clearance are coordinated in tumour antigen‐presenting cells may explain why some tumours convert this debris into productive immunity whereas others develop chronic dysfunctional inflammation.

A second question is how cells avoid responding to self‐nucleic acids in the endolysosomal system. Endogenous RNA modifications such as pseudouridine can reduce recognition by TLR7 and TLR8 [247], limiting inappropriate inflammatory activation. However, how TLR3 responds to endogenous double‐stranded RNA remains unclear, particularly when mitochondrial dsRNA may enter lysosomes under stress. These mechanisms are highly relevant to tumours with mitochondrial dysfunction and to treatments that induce mitochondrial damage.

Immune cells continuously ingest glycan‐rich material from dead cells, microbes and particles, and lysosomal processing determines whether this cargo supports antigen presentation, controlled sensing or pathological escalation. This may be especially relevant in tumours, where extracellular matrix turnover generates abundant glycans and hyaluronan can promote motility and invasion through CD44 signalling [248]. Linking lysosomal glycan processing to inflammatory tone and tissue remodelling could connect clearance biology to tumour progression and metastasis.

Lysosomal immunometabolites may provide additional links between lysosome state and immune fate. As described above, polyamines are endocytosed into lysosomes and require regulated export, for example through ATP13A2 [126, 249]. Polyamine metabolism is essential for lymphocyte proliferation and helper T‐cell differentiation [125].

Another metabolite, pseudouridine, can arise from lysosomal RNA degradation and be released extracellularly, where it may shape tumour microenvironment signalling and support pancreatic tumour development [250]. Whether other immunometabolites are stored, processed, or mobilised through lysosomes remains an open question with implications for therapy response.

Together, these directions may clarify how lysosomes shape immune tone and antigenicity in tumours and identify pathways that can be targeted to improve immunotherapy, reduce immune suppression, or limit tumour‐promoting inflammation.

## 6 Conclusions

This review highlights four lysosomal functions that converge on core oncological problems. First, tumours increase lysosome‐dependent nutrient acquisition through autophagy, endocytosis and macropinocytosis, then rely on efficient hydrolysis and metabolite export to sustain biosynthesis under nutrient limitation. This supports growth in poorly perfused regions, survival during detachment and metastasis, and metabolic buffering during targeted therapy.

Second, lysosomal membranes act as signalling platforms where nutrient cues and glycolytic state tune mTORC1 and AMPK. This allows tumours to coordinate anabolic demand with nutrient availability and links lysosomal stress responses to treatment resistance through altered growth‐factor signalling, redox adaptation and cell‐cycle control.

Third, membrane contact sites position lysosomes within organelle networks, coupling them to ER lipid supply, mitochondrial quality control and spatial proteostasis. These connections can influence invasion, stem‐like states and tolerance to oxidative and proteotoxic stress during cancer progression and therapy.

Fourth, lysosomes shape tumour immunity by supporting antigen processing and presentation, immune‐cell metabolic rewiring and controlled termination of nucleic‐acid‐driven inflammation through nucleases, nucleoside transporters and cGAS–STING degradation. Lysosome state can therefore influence both tumour cell fitness and whether the tumour microenvironment sustains productive immunity, immune suppression or chronic myeloid inflammation.

More broadly, the protein products of many lysosome‐related genes remain poorly defined. Structure‐prediction resources such as AlphaFold can prioritise experimental annotation and may reveal new cancer‐relevant dependencies alongside targets in lysosomal storage disorders.

Lysosomes are also emerging as therapeutic delivery and degradation platforms in cancer. Proteolysis‐targeting chimaeras (PROTACs) generally direct intracellular proteins to the ubiquitin‐proteasome system, whereas lysosome‐targeting chimaeras (LYTACs) can redirect extracellular or membrane‐associated targets towards lysosomal degradation [251, 252].

As introduced above, tumour‐associated PD‐L1 suppresses antitumour T‐cell responses through PD‐1. Because this activity depends on surface PD‐L1, lysosomal degradation of PD‐L1 provides a strategy for disrupting this immune checkpoint [202, 203, 204, 205, 206]. Lysosome‐engaging chimaeras that reduce surface PD‐L1 may complement checkpoint blockade by physically removing the ligand rather than only preventing receptor‐ligand binding [242, 251, 252]. In parallel, lysosome‐targeted vaccine chimaeras may enhance intratumoural antigen handling and presentation [253].

Lysosomes also affect engineered cellular immunotherapy. CAR‐T‐cell cytotoxicity depends on lysosome‐related lytic granules, while CAR internalisation and receptor turnover can involve endosome–lysosome trafficking that influences surface receptor density and serial killing. These processes make lysosomal trafficking an important consideration in the optimisation of cellular cancer therapies.

Together, these advances position lysosomes as increasingly important therapeutic targets and platforms, particularly in cancer immunotherapy.

## 7 Perspectives

The growing dependence of tumours on lysosomal catabolism, signalling and immune regulation provides a basis for a clinically oriented lysosome framework (Fig. 4). This approach could use measurable lysosome‐state outputs, including lysosomal biogenesis and acidity, transporter activity, autophagic flux and inflammatory control, to define tumour‐specific lysosomal dependencies.

**Fig. 4:**
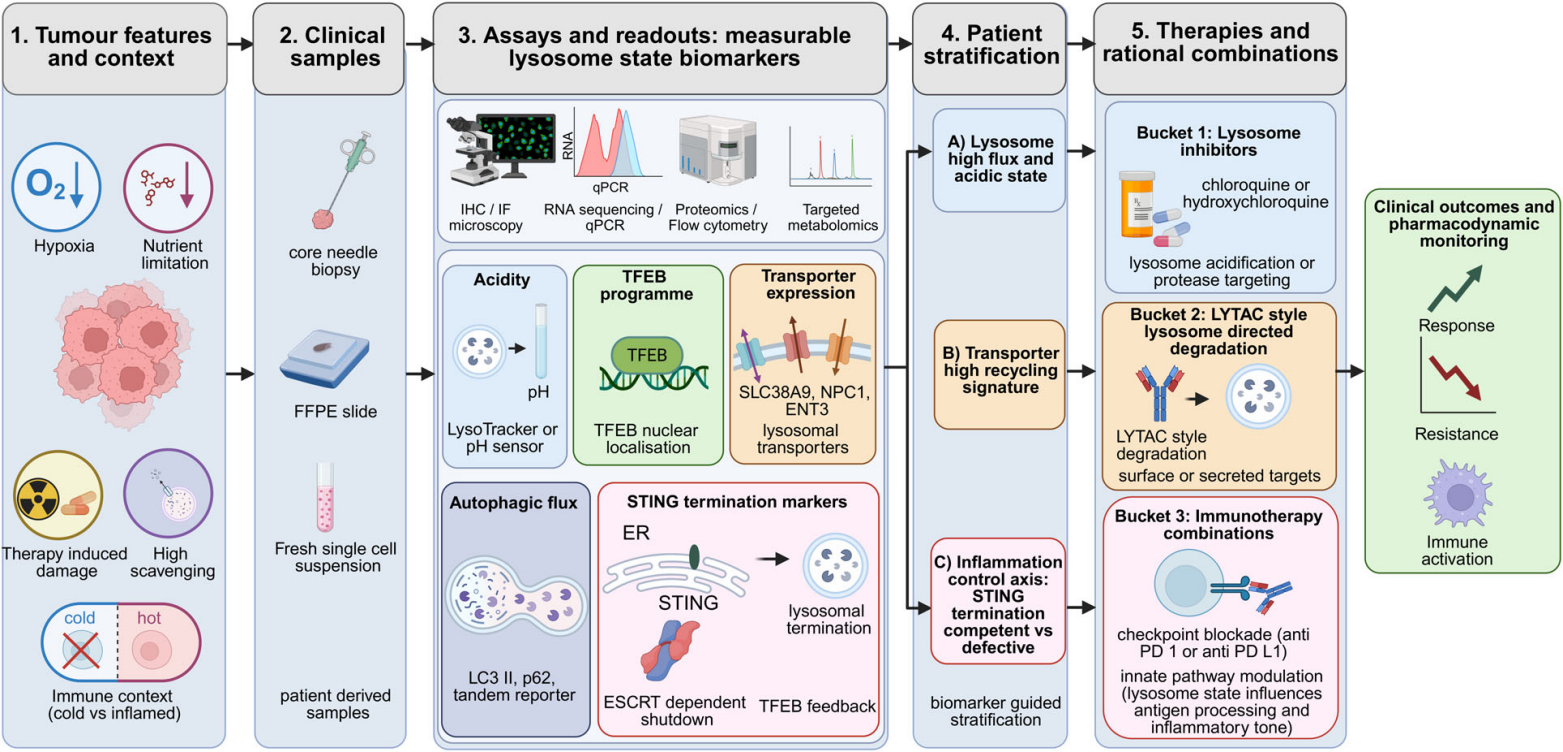
Clinical translation roadmap: measurable lysosome‐state biomarkers and combination strategies. The figure outlines a proposed workflow for translating lysosome biology into cancer biomarkers and treatment strategies. (1) Tumour context: hypoxia, nutrient limitation, therapy‐induced damage, scavenging demand and immune tone may shape lysosome reliance. (2) Clinical samples: core biopsies, FFPE sections, fresh single‐cell preparations or patient‐derived samples. (3) Lysosome‐state readouts: acidity/protease activity, TFEB programmes, transporter expression, autophagic flux and lysosome‐dependent cGAS‐STING termination. (4) Patient stratification: tumours are grouped by biomarker profiles, such as high‐flux acidic states, transporter‐high recycling states, or inflammatory control states defined by STING termination competence. (5) Therapeutic combinations: these profiles may guide lysosome inhibitors (chloroquine and hydroxychloroquine), lysosome‐targeting chimaera (LYTAC)‐style lysosome‐directed degradation and immunotherapy (PD‐1/PD‐L1) combinations, with pharmacodynamic monitoring of response, resistance and immune activation. Abbreviations: FFPE, formalin‐fixed, paraffin‐embedded; LYTAC, lysosome‐targeting chimaera; PD‐1, programmed cell death protein 1; PD‐L1, programmed death‐ligand 1; STING, stimulator of interferon genes; TFEB, transcription factor EB.

Such readouts could support patient stratification into states such as high‐flux acidic tumours, transporter‐high recycling tumours or inflammatory control states defined by STING termination competence. These profiles could then guide rational therapeutic combinations, including lysosome inhibitors such as chloroquine and hydroxychloroquine, lysosome‐targeting chimaera (LYTAC)‐based degradation strategies and PD‐1/PD‐L1 immunotherapy, with pharmacodynamic monitoring of response, resistance and immune activation.

Overall, linking tumour context and clinical samples to lysosome‐state biomarkers, therapeutic strategies and response monitoring may help convert lysosome into a tractable therapeutic axis targeting tumour cell fitness and antitumour immunity together.

## Conflict of interest

The authors declare no conflict of interest.

## Author contributions

JL and KMR conceived and designed the review. JL performed the literature search, wrote the first draft and prepared the figures. KMR critically reviewed and edited the manuscript for important intellectual content. Both authors approved the final manuscript.

## Data Availability

Data sharing is not applicable to this article as no new datasets were generated or analysed in this study.
